# LKB1信号通路在非小细胞肺癌相关靶向治疗中的研究进展

**DOI:** 10.3779/j.issn.1009-3419.2012.07.08

**Published:** 2012-07-20

**Authors:** 竞 王, 瑞丽 韩, 殿胜 钟

**Affiliations:** 1 300052 天津，天津市肺癌研究所 Tianjin Lung Cancer Institute, Tianjin 300052, China; 2 300052 天津，天津医科大学总医院呼吸科 Department of Respriatory Medicine, Tianjin Medical University General Hospital, Tianjin 300052, China

肺癌的发病率和死亡率在全球恶性肿瘤中位于首位，其中，非小细胞肺癌（non-small cell lung cancer, NSCLC）占80%-85%。绝大多数NSCLC临床确诊时处于复发或转移性的晚期阶段，化疗是主要的治疗手段。但是，作为标准一线治疗的含铂类药联合化疗方案已进入平台期，近些年来，分子靶向治疗研究使人们看到了跨越这一平台的希望，是现在肺癌治疗的热点和趋势。

肿瘤可以认为是一种基因疾病，其发生、发展过程中最为关键的环节之一就是癌基因的激活和抑癌基因的失活。近年来，一个新的抑癌基因*LKB1*，又名*STK11*，在肿瘤中的作用引起了很多的关注。在NSCLC中，*LKB1*的突变率可高达15%-35%^[1]^。哺乳动物雷帕霉素靶蛋白（mammalian target of rapamycin, mTOR）信号通路是目前肿瘤信号传导通路研究的热点之一。研究^[1]^显示，LKB1通过磷酸化磷酸腺苷激活的蛋白激酶（AMP activated protein kinase, AMPK），从而激活AMPK，实现对mTOR活性的负向调控，进而通过缺氧诱导因子1（hypoxia inducible factor 1, HIF1）负向调控其下游的赖氨酰氧化酶（lysyl oxidase, LOX）及LOX下游的局部粘着斑激酶（focal adhesion kinase, FAK）；此外，LKB1能够抑制癌促活化因子3抗体（polymoma virus enhancer-3, PEA3）的表达，并最终导致环氧化酶-2（cyclooxygenase-2, COX-2）mRNA和蛋白表达的减少（图 1）。由于*LKB1*是一个抑癌基因，所以其本身并不是一个理想的药物靶点，但其信号通路的下游蛋白已成为治疗的潜在靶点，本文将概述近年来的研究进展。

**1:**
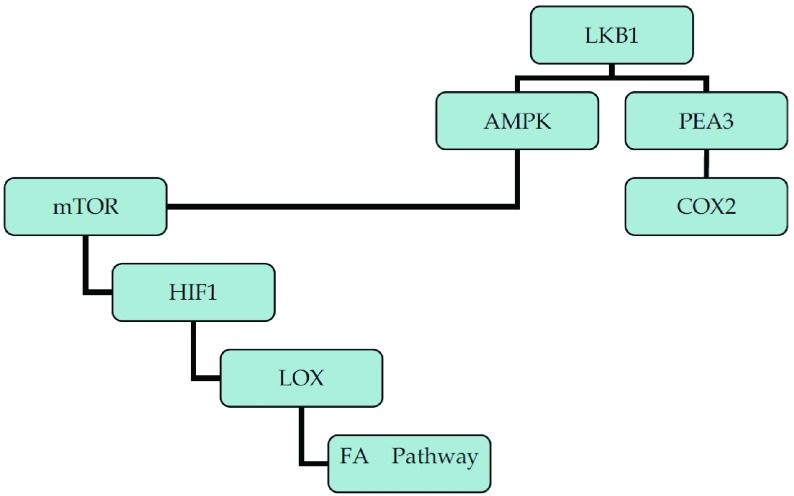
LKB1及其下游相关靶点信号通路示意结构图 Schematic illustration of LKB1 signaling pathway and related downstream targets for cancer therapy

## 1 针对AMP激活的蛋白激酶（AMP-activated protein kinase, AMPK）的靶向治疗

AMPK介导了从LKB1到mTOR-HIF1α的信号通路，研究^[2]^显示*LKB1*基因缺失的细胞一般会失去其下游AMPK靶点对AMPK激动剂的反应性，因此能够不依赖于LKB1而激活AMPK的化合物，诸如二甲双胍、苯乙双胍以及间苯二甲酸（PIA）可能都对肺癌治疗有着比较好的效果^[3, 4]^。这类药物可以不依赖体内的LKB1而激活AMPK，这一作用主要是通过CaMkkβ介导的，因此能够应用于*LKB1*突变的肿瘤患者的治疗^[2]^。这类药物广泛的生物学效应主要体现在能够独立的影响AMPK、AKT和P38α的激活，由此揭示了它们作为抗肿瘤药物的潜力。多项研究^[5-9]^报道了二甲双胍对乳腺癌、前列腺癌、卵巢癌以及结直肠癌均有明显的抑制作用。武宁等^[10]^也发现，二甲双胍对于人类肺腺癌A549细胞具有较强的生长抑制作用。

## 2 针对mTOR的靶向治疗

mTOR是一种丝/苏氨酸蛋白激酶，广泛存在于酵母和哺乳动物中，进化十分保守。mTOR是1991年Heitman等在分析不同啤酒酵母突变体对雷帕霉素抵抗作用差别时发现的。mTOR对生长因子、胰岛素、营养物质、氨基酸、葡萄糖等刺激产生应答，在调节细胞生长、增殖、调控细胞周期等多个方面扮演着重要角色^[1]^。研究表明，mTOR抑制剂通过阻断细胞周期^[11]^、促进肿瘤细胞发生凋亡和自噬并抑制肿瘤血管的生成^[12]^，从而达到抑制肿瘤生长和进展的作用。

由于LKB1-AMPK信号通路下游的mTOR在LKB1缺失的肿瘤细胞中异常活跃，因此mTOR抑制剂被认为在肺癌的治疗中有明显的优势。目前，已有4种mTOR抑制剂被有效地应用于临床，分别是雷帕霉素和3种雷帕霉素的衍生物：CCI-779、RAD001和AP23573。雷帕霉素是一种mTOR抑制剂，最初用于抗真菌治疗，后来又作为免疫抑制剂。Markus等^[13]^在动物实验中发现，雷帕霉素不仅在免疫抑制效果上比环孢素A强10倍以上，而且作为一种非细胞毒性药物，对肿瘤有抑制作用。Mahoney研究^[14]^表明，*LKB1/KRAS*联合突变的肺癌患者表现出了对MEK抑制剂CI-1040的敏感性，同时也表现出了对雷帕霉素的敏感性，但是单独突变的患者对雷帕霉素不敏感，因此，mTOR抑制剂可作为*LKB1/KRAS*联合突变肺癌患者的靶向治疗。研究^[15]^表明，mTOR抑制剂无论是单独用药还是和其它化疗药联合用药，均有很好的抗肿瘤活性。此外，mTOR抑制剂联合磷酸肌醇3激酶（phosphatidylinositol-3-kinase, PI3K）或MEK1/2抑制剂，也显示出其对LKB1缺失的原发和转移肺癌有着很好的协同治疗效果^[16]^。

雷帕霉素的抗肿瘤活性虽强，但它有两个严重的缺点：稳定性差及溶解性差，即使在生理pH条件下也会发生水解作用导致活性下降，而雷帕霉素的三个衍生物能很好的弥补其不足^[17]^。RAD001是Novartis公司研发的半合成衍生物，其水溶性比雷帕霉素好，具有免疫抑制及抗肿瘤作用，在体内免疫活性与雷帕霉素相当。临床前期研究表明，其与细胞毒药物联合应用时具有相加或协同作用。目前，该药在肿瘤的治疗中已处于Ⅰ期-Ⅲ期临床研究阶段。CCI-779是水溶性更好的丙酸酯类衍生物，几乎无免疫抑制活性，是一种延迟肿瘤增殖的非细胞毒药物。AP23573是由计算机辅助设计出来的半合成药物，在各种有机溶剂、水溶液及全血中都比较稳定，无免疫抑制活性，但具有较强的抗肿瘤作用。这三个雷帕霉素衍生物在静脉给药途径中均有更好的药物动力学效应，其中CCI-779和AP23573可口服和静脉给药，RAD001可口服给药。

## 3 针对赖氨酰氧化酶（lysyl oxidase, LOX）的靶向治疗

LOX能够氧化胶原蛋白中的赖氨酸，导致细胞外基质结构的稳定和共价交联^[18]^。LOX的表达或是酶活性的异常与很多疾病相关，包括肺癌。研究^[19]^显示，肺癌患者中LOX血清活性升高，可作为肺癌预后的生物标志物。

研究^[19]^显示，LOX是LKB1-mTOR-HIFα信号通路下游的一个靶点。β-胺基丙腈（β-aminopropionitrile, BAPN）是LOX的抑制剂，Gao等^[19]^研究发现，BAPN能够有效地抑制*LKB1*基因缺失组小鼠的肿瘤大小和数量，但对LKB1野生型组小鼠不明显；此外，BAPN对*LKB1*基因缺失组小鼠的大型肿瘤作用比对小型肿瘤作用明显。上述结果提示，LOX抑制剂对*LKB1*基因缺失的肺癌有抑制肿瘤增殖和促进肿瘤细胞凋亡的作用。另外，该研究还发现，将敲除了*LOX*基因的A549细胞种植到裸鼠体内，其生成的肿瘤的数量和体积要明显小于对照组，能够明显减缓*LKB1*基因缺失肺癌细胞的恶性程度和侵袭性。这些研究结果提示，LOX可能是一个重要的潜在的肺癌治疗靶点，并有希望成为预测肺癌预后的生物学标志物。

## 4 针对局部粘着斑途经（focal adhesion pathway, FA pathway）的靶向治疗

LKB1缺失可以增加局部粘着斑动力学中的蛋白激酶活性，进而促进细胞的运动性和侵袭力。粘着斑是动态的亚细胞结构，能够调节细胞连接到细胞外基质，它由超过50种蛋白组成，包括局部粘着斑激酶（focal adhesion kinase, FAK）、桩蛋白（Paxillin，是迄今为止发现的惟一能与癌基因结合的含酪氨酸的粘附调节蛋白，主要定位于粘着斑，与多种肿瘤的形成有关）和src基因编码产物（SRC）。SRC和FAK活化通过下调RhoA导致了局部粘着斑的拆卸，进而导致了细胞运动性的增加，即加快了转移进程中的细胞运动性和侵袭力，LKB1下调能够导致局部粘着斑组分的活化，特别是SRC和FAK^[20]^。转移是一个多步骤的过程，包括细胞与细胞外基质交互作用、细胞的迁移以及肿瘤的侵袭，这些过程都可以调节SRC和FAK的活性以及被它们调节。Carretero等^[16]^报道，使用SRC家族激酶抑制剂达沙替尼（dasatinib）和FAK抑制剂PF573228干预LKB1缺失和正常表达的细胞，发现用达沙替尼和PF573228处理LKB1敲除的H358细胞，细胞粘附和剂量相关性与非LKB1敲除细胞相比更明显，对于用这两种药处理的两组细胞的细胞迁移能力的检测也发现了类似的结果。这些结果表明，LKB1表达减少可活化SRC和FAK，促进细胞迁移和粘附到细胞外基质。

达沙替尼是一种ATP竞争性的酪氨酸激酶抑制剂，是SRC家族激酶抑制剂。Carretero研究^[16]^显示，单独应用达沙替尼或BEZ235（一种PI3K-mTOR的抑制剂）/AZD2644（一种MEK1/2的抑制剂）不能对Kras/LKB1联合缺失的肺癌肿瘤起作用，而联合应用达沙替尼与BEZ235和AZD2644，经核磁和病理学证实可以对其产生明显的抑制作用；另外，三种药联合还可同时抑制SRC、TGF-β以及MEK、AKT、EMT和ES的信号传导，减少80%的Kras无突变/LKB1缺失的哺乳动物肺癌模型的肿瘤体积。尽管达沙替尼单独使用在*Kras/LKB1*联合突变的肺癌细胞中不能证明对生存有益，但它仍能阻断转移。研究结果^[21]^已经证实，达沙替尼在肺癌和前列腺癌中有抗恶性细胞增生和防止恶性细胞扩散的作用。一项NSCLC的Ⅱ期临床研究结果^[22]^显示，NSCLC人群中存在潜在的对达沙替尼敏感的亚组。

## 5 作用于环氧化酶-2（cyclooxygenase-2, COX-2）的靶向治疗

COX-2是LKB1信号通路下游的重要中介者，很多恶性肿瘤，包括肺癌中的COX-2表达非常高。大样本研究表明，NSCLC细胞表面的COX-2阳性率较高，其中以腺癌阳性率最高，鳞癌次之。Achiwa^[23]^报道，COX-2表达水平与Ⅰ期肺癌患者的生存率下降相关，COX-2表达高可能对预知早期癌症患者临床外科手术预后有意义。对于COX-2的靶向治疗已经被用于临床前期的动物模型中，实验证据显示，COX-2在肿瘤进展过程中起作用，如过表达的COX-2可抑制细胞凋亡，增强了鼠上皮细胞潜在的致瘤性；COX-2在癌细胞侵袭性和血管内皮细胞化疗反应性等体外实验中扮演了重要的角色。但临床上关于COX-2的作用尚需更大样本的群体性研究。目前认为，COX-2参与肿瘤的发生、发展、转移和预后的机制可能有四个方面：抑制凋亡、抑制机体的抗肿瘤免疫、促进肿瘤新生血管生成、增加侵袭力。

COX-2特异性抑制剂（塞来昔布，罗非昔布）已被报道有抗肿瘤的活性，其在NSCLC中可考虑与化疗和放疗联合应用^[24]^。塞来昔布是研究最多的一种COX-2特异性抑制剂，常常与放化疗联合用于NSCLC的治疗，目前已进入Ⅱ期临床实验^[25]^。在2003年第10届国际肺癌会议上有研究者提出，EGFR酪氨酸激酶抑制剂可以联合COX-2抑制剂治疗NSCLC。O’Byme^[26]^公布了一项进行中的联合吉非替尼（gefitinib）和罗非昔布（rofecoxib）治疗曾接受铂类药物治疗的NSCLC，有效率为9.7%，无3级毒性作用的出现。

## 6 其它

有研究发现，LKB1缺失会改变癌细胞的新陈代谢，缺少LKB1的细胞不能对能量压力做出反应进而导致了细胞死亡，提示那些影响细胞新陈代谢的药物可能有利于肿瘤的治疗。据Inge^[27]^报道，LKB1缺失的NSCLC患者比野生型LKB1的患者对2-脱氧葡萄糖（2-DG）的治疗更加敏感，2-DG是一种糖代谢抑制剂，上述结果证实了诱导能量压力的葡萄糖类化合物可能对那些LKB1缺失的肺癌患者有治疗益处。

综上所述，各种针对LKB1信号通路的靶向治疗都有不同程度的抑制NSCLC肿瘤的作用，这为发展新的NSCLC临床治疗策略指出了方向。
